# Survey data to evaluate consumer behaviour and consumption pattern of sustainable apparel: A study on consumer awareness level

**DOI:** 10.1016/j.dib.2023.109350

**Published:** 2023-06-29

**Authors:** Manish Mishra, Rohit Kushwaha, Nimit Gupta, Amit Sinha, Hemverna Dwivedi

**Affiliations:** a Amity Business School, Amity University Uttar Pradesh, Lucknow, India; b School of Management and Liberal Studies, The NorthCap University, Gurugram, India

**Keywords:** Sustainability, Sustainable apparel, Environmental effect, Consumer awareness

## Abstract

A consumer survey was conducted in Uttar Pradesh in five major cities Lucknow, Kanpur, Agra, Varanasi, and Meerut District, from 2022 to 2023 to understand consumer awareness and consumer behaviour towards Sustainable Apparel. The data were collected from 384 respondents via face-to-face interviews through field surveys in different colleges and universities. A paper-based questionnaire was analyzed using SPSS (version 22) using descriptive and, correlation, ANOVA, and regression statistical analysis.

**Specifications Table**

**Table:** 

Subject	Marketing
Specific subject area	Consumer Behaviour
Type of data	Table
How the data were acquired	A survey using a paper-based questionnaire was used to collect data in the selected city of Uttar Pradesh.
Data format	Raw Data (.xls) Analyzed and Descriptive
Description of data collection	Face-to-face, self-administered questionnaires were distributed to participants in Colleges and Universities The purpose of the research was explained to participants, and their consent was obtained before distributing the survey. The sampling technique used was Random and Purposive in nature, and the research data, questionnaire file, and SPSS file can be found in a repository [18]. The questionnaire is divided into two sections, with Section A focusing on demographic information and Section B consisting of the instruments employed. The data collected is quantitative in nature and includes descriptive statistics. Participants responded using a 5-point Likert scale. A paper-based questionnaire survey was utilized to gather data in the selected city of Uttar Pradesh
Data source location	Lucknow District (India) Kanpur District (India) Meerut District (India) Varanasi District (India) Agra District (India)
Data accessibility	Repository name: Mendeley Data identification number: 10.17632/9rfc9zf3p2.4 Direct URL to data: https://data.mendeley.com/datasets/9rfc9zf3p2/4

## Value of the Data

• The data provides insight on the factors that influence the consumer behaviour of sustainable apparel in India.
• The data in this article can aid policymakers in creating effective strategies for promoting sustainable clothing consumption among consumers.
• The dataset can be used by other researchers to compare with other data acquired from similar studies from other geographically locations or regions.
• The data allows other academicians and researchers to extend the statistical analysis, i.e., structural equation modelling.

## 1 Objective

The objective of this study is to collect and analyze data on consumer behaviour and consumption patterns related to sustainable apparel and to evaluate the level of consumer awareness and knowledge about sustainable clothing. Furthermore, the data will also be used to identify potential gaps in consumer awareness, as well as opportunities for sustainable apparel brands to improve their marketing, product development, and social responsibility initiatives. The findings of this study can contribute to a larger understanding of the intersection between consumer behaviour and sustainability in the fashion industry.

## 2 Data Description

The survey data aims to evaluate the consumer behavior and consumption patterns of sustainable apparel, specifically focusing on the awareness level of consumers. The data was collected through a survey method. The survey consists of demographic information and close -ended questions. A total number of 384 [15] participants completed the survey using a paper-based questionnaire from August,2022 to January 2023. Participants were asked to indicate to the extent which they agree with the statements related to Motivation, Product Knowledge, Environment Knowledge, Perceived Value, Consumer Awareness, Purchase Intention, buying behaviour on 5-point Likert scale, strongly Disagree (1) to Strongly agree (5). The Raw data contains responses from the respondents. The Nominal data coded as Gender-1=Male, 2=Female; Age-1=18 years -24 years, 2=25 years -31 years,3=32 years -38 years, 4=38 years above; highest level of education completed-1=Undergraduate,2=Graduate,3=Post-graduate, 4=Professional; occupation-1=Student,2=Employee,3=Businessman,4=Homemaker,5=Retired, Current location-1=Lucknow,2=Kanpur, 3=Meerut,4=Varanasi,5=Agra; monthly household Income-1=Less than 50,000, 2=50,000-1,00,000, 3=Above 1,00,000 and Measured Items responses are asked to indicate the extent to which you agree or disagree with each statement, each statement, using a 5-point Likert scale (1) = strongly disagree; (2) = disagree; (3) = neutral; (4) = agree; (5) = strongly agree. The data raw (.xlsx), research questionnaire (.docx), scale development (.xlsx), SPSS file (.sav) and Codebook are available in https://data.mendeley.com/datasets/9rfc9zf3p2/4

Table 1 presents the demographic characteristics of the sample based on gender, age, education level, income level, and city. Table 2 provides a summary of the constructs examined in the study, along with their definitions and sources. Table 3 displays the Descriptive statistics and reliability using Cronbach's alpha of the measured constructs.

**Table 1:** Demographic profile of respondents.

Demographic variables Category		Frequency	Percentage
**Gender**	Male	131	34.1%
	Female	253	65.9%
**Age**	18 years -24 years	220	57.3%
	25 years -31 years	136	35.4%
	32 years -38 years	12	3.1%
	38 years above	16	4.2%
**Education Level**	Undergraduate	210	54.7%
	Graduate	24	6.3%
	Post-graduate	120	31.3%
	Professional	30	7.8%
**Occupation**	Student	338	88.0%
	Employee	25	6.5%
	Businessman	12	3.1%
	Homemaker	9	2.3%
	Retired	0	0.0%
**City**	Lucknow	186	48.4%
	Kanpur	64	16.7%
	Meerut	22	5.7%
	Varanasi	48	12.5%
	Agra	64	16.7%
**Household Monthly Income**	Less than 50,000	131	34.1%
	50,000-1,00,000	100	26.0%
	Above 1,00,000	153	39.8%

**Table 2:** Construct, definition, and source.

Construct	Definition	Code	Measurement Items	Source
**Product Knowledge**	Product knowledge refers to the understanding consumers have about a product, including its features, benefits, and production processes. In the context of sustainable apparel, product knowledge includes knowledge about sustainable materials, production processes, and environmental and social impact.	PK1	Clothing made from organic fibers is stylish.	[1]
		PK2	Clothing made from organic fibers has attractive designs.	[1]
		PK3	Clothing made from organic fibers is durable.	[1]
		PK4	Clothing made from organic fibers has good quality.	[1]
**Consumer Awareness**	Consumer awareness is the level of knowledge and understanding that consumers have about a product or service, including its benefits, drawbacks, and impact on the environment and society.	CA1	I am aware that garments made from recycled materials will biodegrade once their useful life is over, instead of adding to a landfill.	[2]
		CA2	I am aware that companies can follow standards of environmentalism and social policies in areas related to the production of goods, including clothing.	[2]
		CA3	I am aware that second-hand clothes or upcycled clothes made by using existing materials can be sustainable choices.	[2]
		CA4	I am aware that products made without the use of animal tissue products are considered more sustainable.	[2]
**Perceived Value**	Perceived value refers to the worth or benefits that a customer believes they will receive from a product or service. It is based on their subjective evaluation of the benefits and costs associated with the product or service.	PV1	I believe that sustainable clothing is well-made and worth the money.	[3]
		PV2	I feel good when I purchase sustainable apparel.	[3]
		PV3	Sustainable clothing offers a unique style to me.	[3]
		PV4	I believe that sustainable clothing helps in saving resources.	[3]
**Motivation**	Motivation refers to the driving force or reason that compels an individual to take action or behave in a certain way.	MOT1	I feel good about contributing to the environment through the practice of sustainable clothing.	[4]
		MOT2	My friends' opinions on Instagram influence my apparel purchase decisions.	[5]
		MOT3	I feel guilty that animals have died because of human beings' consumption habits.	[1]
		MOT	I believe that clothing made from organic fibers is beneficial for my skin.	[6]
**Environment Knowledge**	In the context of sustainable apparel, environment knowledge refers to understanding the environmental impacts of textile and clothing production, as well as the principles and practices of sustainable design, production, and consumption in the fashion industry.	EK1	I am aware that the dyeing and finishing processes used to produce textiles and clothing can result in a significant amount of water waste.	[6]
		EK2	I am aware that the manufacturing process of synthetic or manufactured fibers such as polyester can cause environmental pollution.	[7]
		EK3	I am aware that air pollution can occur during some common dyeing processes of textiles.	[8]
		EK4	I am aware that the cultivation of raw materials for garments, such as cotton, can have a significant environmental impact.	[9]
**Purchase Intention**	Purchase intention refers to the likelihood or willingness of an individual to buy a product or service in the future. It is influenced by a variety of factors such as perceived benefits, product attributes, price, brand image, and social norms.	PI1	I am willing to pay more for clothing from environmentally and/or socially responsible clothing brands.	[10]
		PI2	In the future, I intend to purchase environmentally sustainable apparel.	[11]
		PI3	I would consider buying environmentally friendly apparel because it is less polluting.	[12]
		PI4	I would consider switching to environmentally friendly apparel brands for ecological reasons.	[12]
**Buying Behaviour**	Buying behaviour refers to the process and actions that individuals or groups go through to purchase goods or services. It includes the decision-making process, the factors that influence the purchase, the actual purchase itself, and the post-purchase evaluation. Buying behaviour is influenced by a wide range of factors such as personal preferences, social and cultural influences, psychological factors, and situational factors.	BB1	When I have a choice between two similar clothing items, I prefer to purchase the one made from textile materials that are less harmful to the environment.	[1]
		BB2	I prioritize selecting apparel that can be worn over a longer term compared to trendy apparel that goes out of style quickly.	[13]
		BB3	I prefer to buy clothing made of organically grown natural fibers.	[13]
		BB4	I prefer to buy apparel that has environmentally friendly labelling or packaging techniques.	[13]

**Table 3:** Mean, standard deviation and reliability of measured constructs.

	Product Knowledge (PK)	Consumer Awareness (CA)	Environment Knowledge (EK)	Perceived Value (PV)	Motivation V (MOT)	Purchase Intention (PI)	Buying Behaviour (BB)
Mean	3.5729	3.4900	3.5368	3.5742	3.4512	3.6263	1.13672
Standard Deviation	80830	1.05483	1.02048	1.06413	.92793	3.5916	1.14548
Cronbach's alpha	0.851	0.873	0.913	0.930	0.870	0.953	0.963

## 3 Experimental Design, Materials and Methods

This research paper used a quantitative approach to collect data on consumer behaviour and sustainable apparel consumption patterns [8,16]. The data was collected using survey questionnaires that included 28 measurement items for the seven latent constructs being studied [13]. The items were adapted from past literature [1], [2], [3], [4], [5], [1], [6], [7], [8], [9], [10], [11], [12], to develop the questionnaire, we employed a search strategy tailored to Scopus and WOS, using the keyword "Sustainable apparel" to identify relevant literature. The searches encompassed articles, review papers, and research reports published in English only, from the database's inception until 2022. The selection criteria were based on the PRISMA Statement [17], focusing primarily on mapping the existing literature on sustainable apparel in the fields of Social Sciences, Environmental Science, Business, and Management. The respondents rated the items on a 5-point Likert scale.

The researchers first identified 10 colleges and universities located in 5 different cities in Uttar Pradesh to collect data on consumer behaviour and consumption patterns of sustainable apparel. From these institutions, a list of potential participants, including students, teachers, and professors, was created. To ensure that the sample was representative of the population under investigation, the researchers used a random sampling method to select participants from this list. Once the participants were selected, the researchers distributed survey questionnaires to gather data. The combination of purposive sampling and random selection helped to minimize potential sources of bias in the selection process. Purposive sampling allowed for the targeted selection of participants based on specific criteria, while a random selection of individuals within these groups helped to ensure that the sample was representative of the population under investigation.

## 4 Ethics Statements

The study utilized nonexperimental voluntary surveys, and the researchers ensured that they adhered to all ethical considerations throughout the data collection process. The researchers obtained consent from the participants before conducting the surveys. The research was carried out in a setting where ethical approval was not mandatory for survey studies. Additionally, the participants' identities were protected as no personally identifiable information was sought.

## Funding

This research did not receive any specific grant from funding agencies in the public, commercial, or not-for-profit sectors.

## CRediT authorship contribution statement

**Manish Mishra:** Writing – original draft, Conceptualization, Formal analysis, Methodology. **Rohit Kushwaha:** Conceptualization, Methodology, Writing – review & editing, Formal analysis. **Nimit Gupta:** Formal analysis, Conceptualization, Methodology, Writing – review & editing. **Amit Sinha:** Methodology, Writing – review & editing. **Hemverna Dwivedi:** Resources.

## Conflict of Interest

The authors declare that they have no known competing financial interests or personal relationships that could have appeared to influence the work reported in this paper.

## Data Availability

Survey data to evaluate consumer behaviour and pattern of consumption of sustainable apparel: A study on consumer awareness level (Original data) (Mendeley Data).
